# Reorganization of mammalian body wall patterning with cloacal septation

**DOI:** 10.1038/s41598-017-09359-y

**Published:** 2017-08-23

**Authors:** Margaret I. Hall, José R. Rodriguez-Sosa, Jeffrey H. Plochocki

**Affiliations:** 1 Department of Anatomy, Arizona College of Osteopathic Medicine, Midwestern University, Glendale, AZ 85308 USA; 2 Department of Anatomy, College of Veterinary Medicine, Midwestern University, Glendale, AZ 85308 USA

## Abstract

Septation of the cloaca is a unique mammalian adaptation that required a novel reorganization of the perineum–the caudal portion of the trunk body wall not associated with the hindlimb. Fish, the basal vertebrates, separate ventrolateral body wall musculature of the trunk into two discrete layers, while most tetrapods expand this pattern in the thorax and abdomen into four. Mammals, the only vertebrate group to divide the cloaca into urogenital and anorectal portions, exhibit complex muscle morphology in the perineum. Here we describe how perineal morphology in a broad sample of mammals fits into patterning of trunk musculature as an extension of the four-layer ventrolateral muscular patterning of the thorax and abdomen. We show that each perineal muscle layer has a specific function related to structures formed by cloacal septation. From superficial to deep, there is the subcutaneous layer, which regulates orifice closure, the external layer, which supplements both erectile and micturition function, the internal layer, which provides primary micturition and defecation regulation, and the transversus layer, which provides structural support for pelvic organs. We elucidate how the four-layer body wall pattern, restricted to the non-mammal tetrapod thorax and abdomen, is observed in the mammalian perineum to regulate function of unique perineal structures derived from cloacal septation.

## Introduction

**Figure 1:**
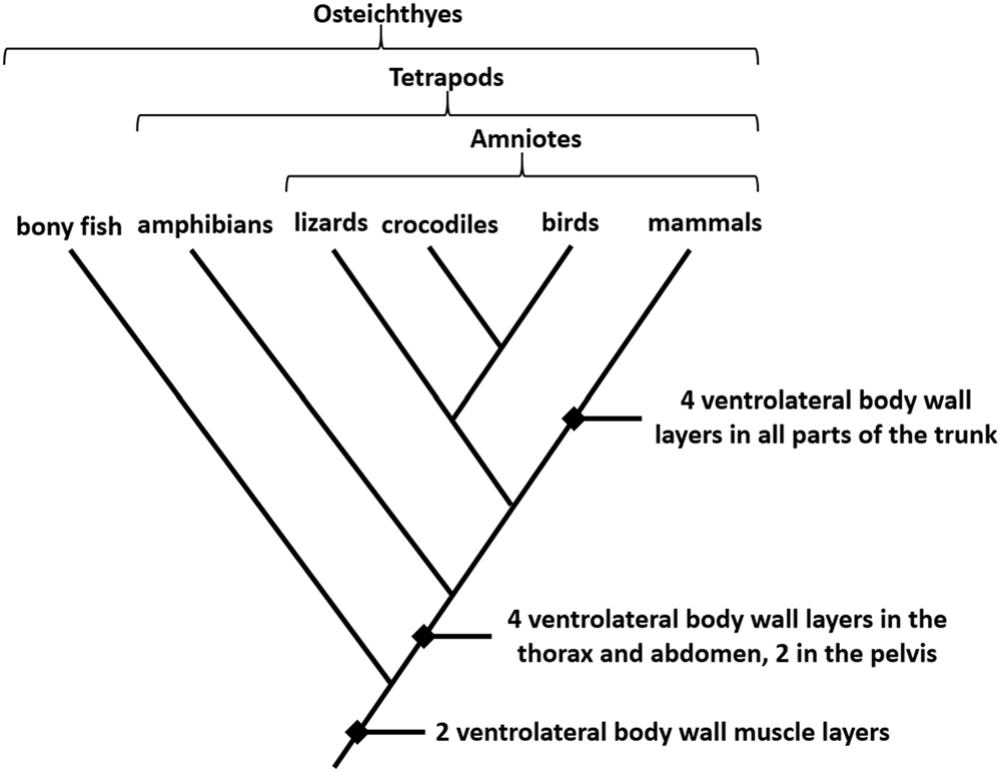
Cladogram of major vertebrate classes demonstrating the evolution of ventrolateral body wall muscle layers. Primitively, vertebrates have two ventrolateral muscle layers, while most tetrapods have four layers in the thorax and abdomen. Mammals exhibit four layers throughout the trunk, including the perineal portion of the body wall.

**Figure 2:**
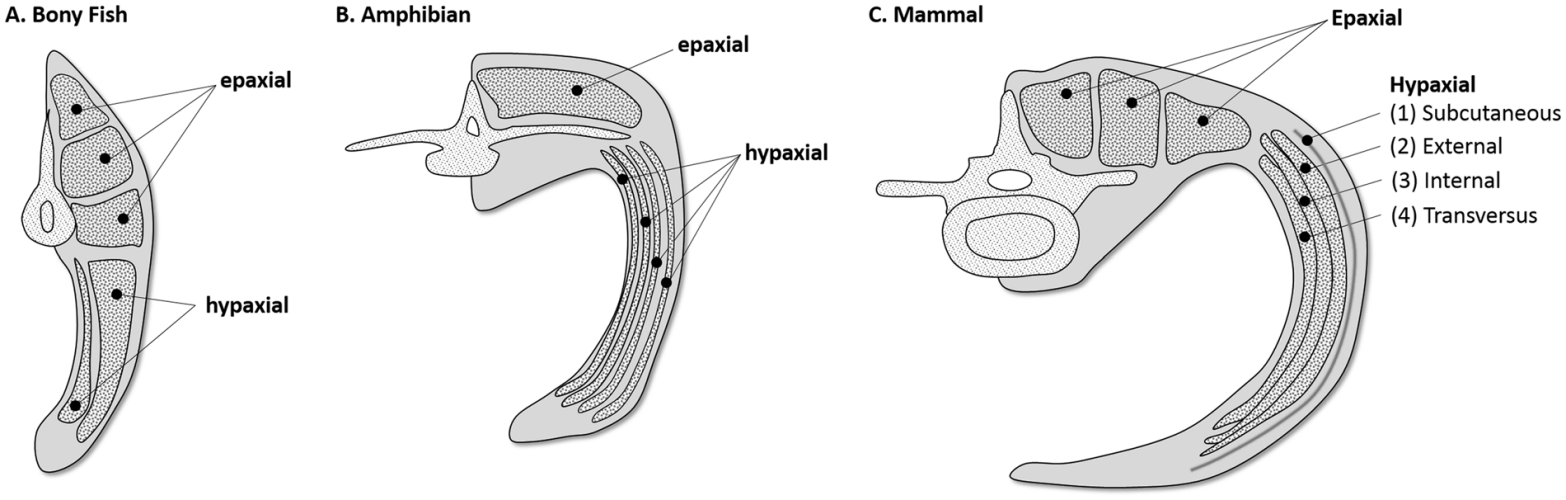
Muscles of the ventrolateral body wall in the vertebrate abdomen derived from the embryonic hypaxial and epaxial masses. (**A**) In fishes, such as teleosts, hypaxial muscles of the ventrolateral wall are organized into two layers (*M. obliquus superioris* and *M. obliquus inferioris*). (**B**) Amphibians, such as salamanders, have two to four layers (*M. obliquus externus*, in some species divided into *superficialis* and *profundus* portions, *M. obliquus internus*, and *M. transversus abdominis*, with scattered subcutaneous skeletal muscle fibers). (**C**) Mammals, like dogs, consistently have four layers (*M. cutaneous trunci*, a well-developed subcutaneous muscle layer, *M. obliquus externus abdominis*, *M. obliquus internus abdominis* and *M. transversus abdominis*). Development of the ventral musculature, such as *M. rectus abdominis*, is regulated by different molecular signals and therefore follows a different organizational pattern and may not differentiate into multiple layers^12^.

Homologous body wall layers that support the vertebrate trunk follow generalized plans that are evolutionarily conserved and broadly shared among related taxa^1, 2^. Basal vertebrates, such as teleost fishes, have ventrolateral wall musculature differentiated into two layers (Figs 1 and 2) that function to laterally flex the trunk during undulatory swimming^3^. With the transition from water to land, morphological complexity of tetrapod ventrolateral body wall musculature in the thorax and abdomen increased as muscle function shifted to include trunk stabilization against torsion and movements of the limbs during terrestrial locomotion^4–6^. Some basal tetrapods, such as certain salamanders and frogs, retain two layers throughout the trunk, while other amphibians developed as many as four layers in the thorax and abdomen^7, 8^. With the rise of amniotes, which include lizards, crocodiles, birds, and mammals, the ventrolateral body wall evolved to consistently maintain four layers in the thorax and abdomen^9–12^.

Most tetrapods retain the original two vertebrate muscle layers present in fishes to regulate opening of the cloacal orifice^13, 14^. Muscular patterning of the pelvic body wall in mammals, which completely separate the cloaca to form the anatomical region of the trunk known as the perineum, has yet to be discussed within an evolutionary context^15–17^. Perineal muscles in mammals develop between the hindlimb bud and tail, receive innervation from spinal levels caudal to the hindlimb, attach to axial and pelvic skeletal elements, and support erectile, defecatory, and micturatory functions of the perineum^18^. Using a comparative approach, we investigate muscle patterning in the perineal portion of the mammalian trunk body wall to gain insight into how muscular reconfiguration in the perineum compares to the primitive vertebrate body plan. We suggest the muscular pattern for the body wall present in the tetrapod thorax and abdomen is utilized again in mammals to regulate function of structures derived from cloacal septation.

## Results and Discussion

**Figure 3:**
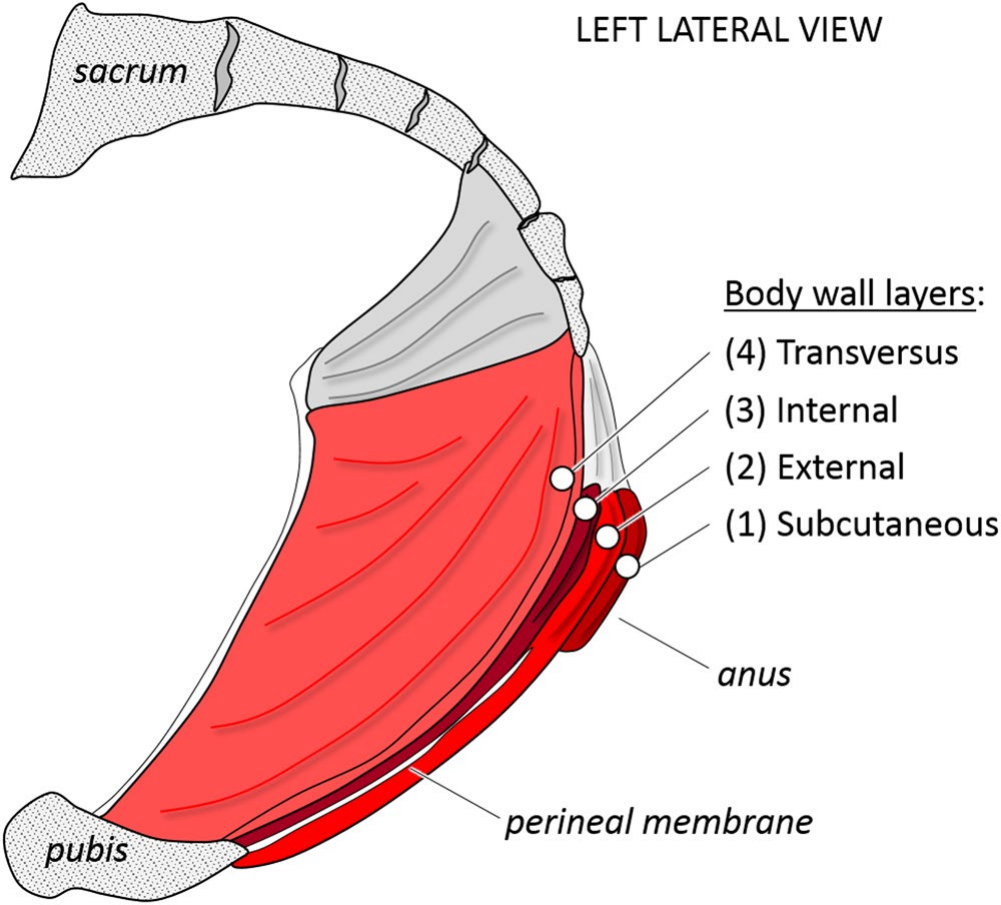
Anatomical organization of the perineal portion of the mammalian trunk body wall into four layers, drawn here after our human dissections. The subcutaneous layer is formed by *M. sphincter ani externus pars subcutanea*. The external layer includes *M. bulbospongiosus*, *M. ischiocavernosus* and *M. transversus perinei superficialis*. The internal muscle layer is formed by *M. sphincter ani externus pars profundus*, *M. transversus perinei profundus*, and the urethral sphincter muscles. The transversus layer includes *M. levator ani* and *M. coccygeus*.

**Figure 4:**
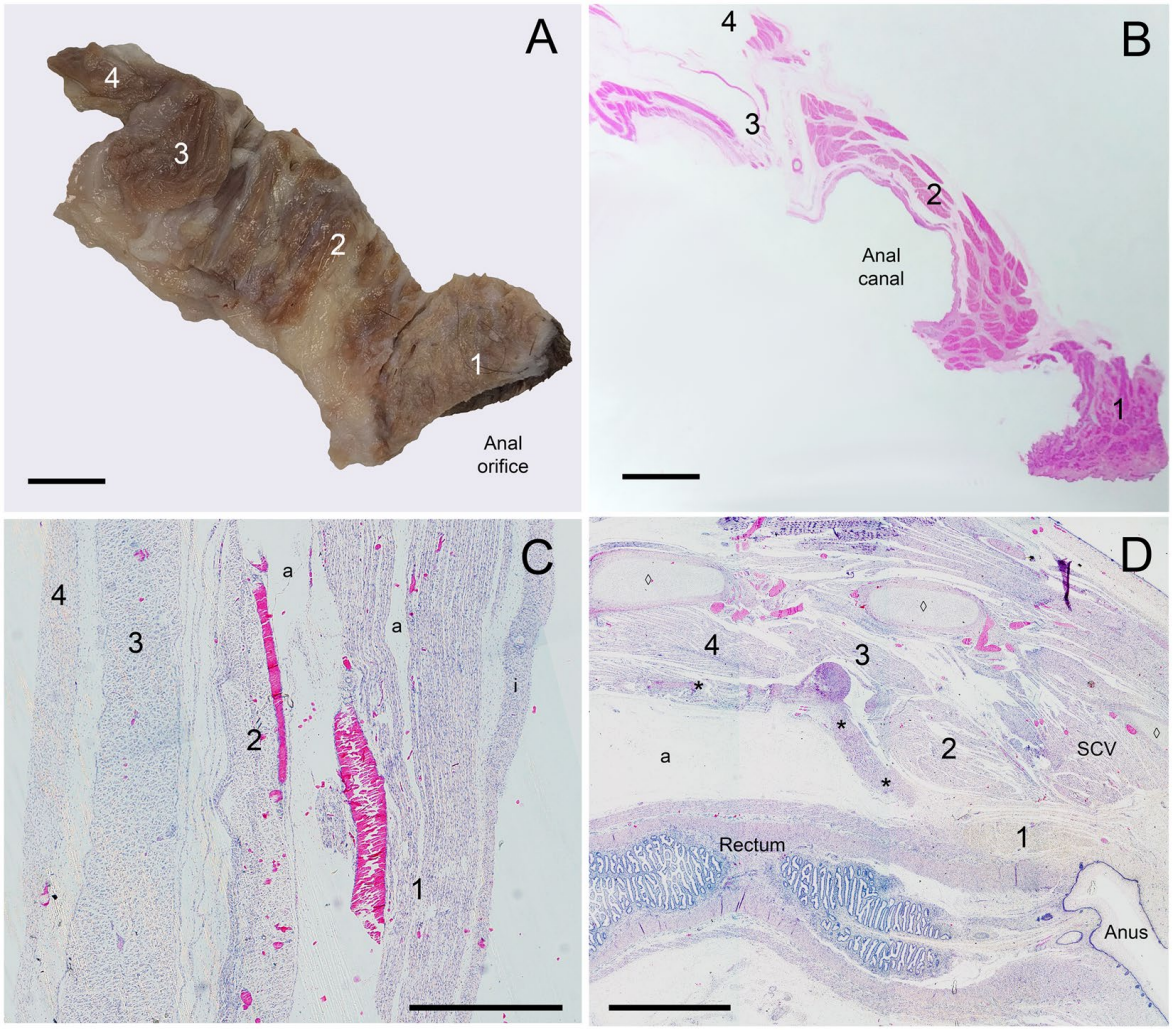
Perineum of an adult dog in (**A**) gross dissection (left lateral view, scale bar 5 mm) and (**B**) histological section (left lateral view, scale bar 5 mm). Histological sections of a fetal bovine aged 15 weeks of the (**C**) ventrolateral abdominal wall (left anterior view, scale bar 50 µm) and (**D**) perineum (left lateral view, scale bar 2 mm). Structures labeled 1–4 are 1, subcutaneous layer; 2, external layer; 3, internal layer; and 4, transversus layer. Perineal muscle fibers (**B** and **D**) of the subcutaneous layer (1) are shown in cross-section, fiber orientation in the internal and external layers (2 and 3) is oblique, and fiber orientation in the transversus layer (4) is longitudinal in the taxa that have an anus that is shifted to protrude posteriorly, such as the bovine, dog, and horse, but is transverse in other taxa. The fetal muscle layers (**D**) are closely associated with smooth muscle of the internal anal sphincter and rectum (*) and developing sacral vertebrae (◊). a, artifact; i, integument; SCV, *M. sacrocaudalis ventralis*, a muscle that acts on the tail.

Our dissections reveal that mammals differentiate pelvic body wall muscles into four layers, mirroring the tetrapod thoracic and abdominal body wall (Figs 2 and 3). These layers include a subcutaneous, external, internal, and transversus layer, with muscles in each layer demonstrating a different fiber orientation (Fig. 4). The perineal muscle layers and associated fasciae are continuous with those of the abdomen and thorax. Subcutaneous muscles of the perineum are present in the same fascial layer as *M. cutaneous trunci* in the thorax and abdomen. Fibers of the external layer insert caudally onto an aponeurosis that is continuous with that of the external layer of the abdomen, *M. obliquus externus abdominis*, which is continuous with *M. intercostales externi* in the thorax. Neurovasculature in the pelvic body wall courses between the internal and transversus layers (e.g., within the pudendal canal), a pattern observed in the thorax and abdomen where neurovasculature courses between *M. intercostales interni* and *M. intercostales intimi*, and *M. obliquus internus abdominis* and *M. transversus abdominis*, respectively. Endopelvic fascia covering the internal surface of the transversus layer of the pelvis is continuous with transversalis fascia of the abdomen and endothoracic fascia of the thorax.

The perineal subcutaneous layer includes the *M. sphincter ani externus pars subcutanea* and *M. constrictor vulvae* (variably present) in mammals we dissected (Fig. 3). The external layer is comprised of the muscle sheet associated with the phallus, which is traditionally divided into *M. bulbospongiosus*, *M. ischiocavernosus* and *M. transversus perinei superficialis*. *M. sphincter ani externus pars superficialis* is comprised of fibers of the *M. bulbospongiosus* portion of the muscle sheet that continues dorsally, continuous with *M. sphincter ani externus pars superficialis*, to form a muscular ring. The internal muscle layer is formed by *M. sphincter ani externus pars profundus* and its continuity with *M. levator ani*. The *M. transversus perinei profundus*, only consistently present in males, is also part of the internal layer and its fibers intermingle with those of *M. sphincter ani externus pars profundus*. This layer also includes the urethral sphincter muscles. The transversus layer is comprised of *M. levator ani* and *M. coccygeus*. The portion of *M. levator ani* most closely associated with the anal canal, *M. puborectalis*, is continuous with the deepest edge of *M. sphincter ani externus pars profundus*. These findings suggest *M. sphincter ani externus* is not a single muscle, but rather a composite of several muscle layers^19, 20^. Because of the notable continuities between *M. levator ani* and *M. sphincter ani externus*, these muscles have also been described as continuous with one another, and even as a single, multi-layered muscle^21–23^.

Histological sections of the perinea of the adult dog and bovine fetus 15 weeks gestation age similarly demonstrate four layers of the ventrolateral abdomen and perineal body wall (Fig. 4). These findings agree with other histological studies of perineal development that show embryological continuities among muscles in each of the four trunk layers and separation of the muscle layers by fascial layers during fetal development^24, 25^. Human fetuses at roughly 13 weeks of development show continuities between the external muscle layer containing *M. ischiocavernosus*, *M. bulbospongiosus*, and the *pars superficialis* of the developing *M. sphincter ani externus*, just inferior to *M. levator ani* of the transversus layer^26–28^. This relationship arises shortly after the cloacal membrane obliterates during urogenital septation and remains in the adult^29, 30^. At this time, *M. sphincter ani externus* surrounds the anal canal, forming a column extending to the ectoderm-derived epithelium^30^. Also around the same time in development, the urinary sphincter muscles become well defined in the internal layer, positioned inferior to the inferior margin of *M. levator ani* of the transversus layer but superior to *M. bulbospongiosus* of the external layer^31^. Separations between muscles in each layer form later in development via apoptosis of muscle fibers^32^. Thus, the muscle layering found in dissection appears histologically subsequent to embryologic septation of the cloaca.

The primitive vertebrate characteristic of two ventrolateral body wall muscle layers was expanded in number and in distribution in the trunk multiple times in vertebrate evolution: at the tetrapod node to four layers in the thorax and abdomen, and again at the mammal node to four layers throughout the trunk, including the perineum (Fig. 1). Mammalian perineal muscular patterning is associated with the complete septation of the cloaca, which led to novel anatomical adaptations in mammals relative to other vertebrates. Specifically, mammals alone among vertebrates evolved the suite of characteristics from the anorectal and urogenital chambers formed by cloacal septation including the rectum, anal canal, urethra, and paired vascular erectile tissues. Other vertebrate intromission structures, such as those variably found in some fish, lizards, turtles, crocodilians, and waterfowl, arise from the cloacal wall and have either a single vascular erectile body or one engorged with lymph, and are not homologous to mammalian genitalia^33–35^. Lizards, crocodilians, birds, and even monotremes have muscular sphincters that regulate opening of the cloaca^9, 11, 36^. These cloacal muscles, as well as perineal muscles in mammals, were proposed to evolve from abdominal trunk muscles in previous studies^37, 38^. However, anatomic, embryonic, and molecular investigations indicate that, while cloacal muscle precursors temporarily reside in the limb, they later migrate to the caudal trunk where they are subjected to the same developmental signaling that regulates trunk muscular layering to form an extended myotomal sheet similar to that of developing trunk body wall muscles^10, 25, 39–42^. This may explain why congenital malformations affecting ventrolateral abdominal body wall musculature are commonly accompanied by perineal muscular defects^43, 44^.

Evolutionary restructuring of mammalian perineal musculature may be a consequence of interactions between muscle precursors and connective tissue during musculoskeletal patterning. Skeletal muscles originate from undifferentiated tissue known as the epaxial and hypaxial masses, which give rise to dorsal and ventral body wall muscles, respectively. Hypaxial muscle cell precursors from the ventrolateral lip of somitic myotomes migrate through the lateral somitic frontier, i.e., from the primaxial mesodermal domain into the abaxial mesodermal domain, to populate the ventral body wall, where they are influenced by connective tissue derived from the lateral plate^45, 46^. Developmental signals expressed in lateral plate mesoderm that result in layering of the abdominal wall also control muscle layering in the perineum, discrete from hindlimb signaling, and are further directed by local signals that fine-tune muscle development^25, 42, 47^. Variations in these interactions, along with the modular property of mesodermal domains, have been used to explain regional differences in anatomic musculoskeletal structure across related taxa^48, 49^. Such anatomic restructuring may also explain similarities of perineal muscle layering with that of the abdomen and thorax, as cloacal sphincter muscles adapted to specialized reproductive and excretory functions in mammals. With the evolutionary establishment of the mammalian perineum, somatic musculature associated with the derived perineal structures was reconfigured into four layers^16^. From superficial to deep, these four muscle layers are (1) the subcutaneous layer, which regulates orifice closure, (2) the external layer, which supplements both erectile and micturition function, (3) the internal layer, which provides primary micturition and defecation regulation, and (4) the transversus layer, which provides structural support for pelvic organs (Fig. 3)^50^.

Our research defines for the first time the four serially homologous trunk ventrolateral body wall layers in the perineum. Our sample consists of primates and domestic mammals that represent a broad distribution of placental mammalian groups that are not closely related phylogenetically, including perissodactyls (horse), artiodactyls (cow, goat, pig), carnivorans (cat, dog) and primates (human, monkey, prosimian) and form a robust sample from which to draw conclusions about perineal morphology^14^. We suggest the primitive anatomical building blocks and developmental signaling used to construct the thoracic and abdominal trunk body wall were repurposed in the perineum. Mammalian perineal structure derived from cloacal septation is an evolutionary innovation that allows for myriad anatomical configurations, diverse reproductive strategies, and precise excretory control available only to mammals.

## Methods

We dissected pelvic and perineal musculature in a subset of adult mammals (Table 1). This sample was obtained from Midwestern University teaching collections, the Arizona Research Collection for Integrative Vertebrate Education and Study (ARCIVES) housed at Midwestern University (Glendale, AZ, USA), and donated human cadavers from the National Body Donor Program (St. Louis, MO, USA). All animals in the study were treated in accordance with the Guide for the Care and Use of Laboratory Animals of the National Institutes of Health with approval from the Institutional Animal Care and Use Committee at Midwestern University. All human cadavers were obtained and studied with informed consent and treated in accordance with local and national laws and regulations with approval from Midwestern University. All muscles of the perineum were dissected according to methods described in Hall and Walters^51^. We identified the following perineal muscles during dissection: *M. coccygeus, M. levator ani*, *M. bulbospongiosus*, *M. ischiocavernosus*, *M. transversus perinei superficialis* and *profundus*, *M. sphincter ani externus* and its subdivisions, and urinary sphincters. During dissection, we observed the layering, attachment and innervation of each muscle. Histological sections 5 µm thick were taken from the perineum and abdomen of an adult dog and a fetal cow aged 15 weeks of an approximately 40-week gestation period. Sections were stained with hematoxylin and eosin and imaged under light microscopy.

**Table 1:** Sample used in our study.

Family	Species	Common name	N
Hominidae	*Homo sapiens*	Human	43
Callitrichidae	*Saguinus oedipus*	Cotton-top tamarin	2
Indriidae	*Propithecus verreauxi*	Verreaux’s sifaka	2
Lemuridae	*Lemur catta*	Ring-tailed lemur	1
Lorisidae	*Perodicticus potto*	Potto	1
Equidae	*Equus caballus*	Horse	3
Bovidae	*Capra hircus*	Goat	9
Bovidae	*Bos taurus*	Cow	1
Suidae	*Sus domesticus*	Pig	9
Canidae	*Canis familiaris*	Dog	21
Felidae	*Felis catus*	Cat	6
Gallidae	*Gallus domesticus*	Chicken	1
